# Long-term pelvic floor symptoms and urogenital prolapse after hysterectomy

**DOI:** 10.1186/s12905-023-02286-3

**Published:** 2023-03-21

**Authors:** Carolien K. M. Vermeulen, Joggem Veen, Caroline Adang, Anne Lotte W. M. Coolen, Sanne A. L. van Leijsen, Marlies Y. Bongers

**Affiliations:** 1 Department of Gynecology and Obstetrics, Máxima Medical Centre, De Run 4600, 5500 MB Veldhoven, The Netherlands; 2 GROW, Research School of Oncology and Developmental Biology, University of Maastricht, Universiteitssingel 40, 6229 ER Maastricht, The Netherlands; 3 Woman Care, Bergman Clinics, Professor Bronkhorstlaan 10, 3723 MB Bilthoven, The Netherlands; 4 Department of Gynecology and Obstetrics, Maastricht University Medical Center, P. Debyelaan 25, 6229 HX Maastricht, The Netherlands

**Keywords:** Hysterectomy, Prolapse, Pelvic floor, Pelvic organ prolapse, Symptoms, Women

## Abstract

**Background:**

The aim of this study was to describe the natural course of pelvic floor symptoms and pelvic floor anatomy for women long-term after hysterectomy.

**Methods:**

Women who underwent hysterectomy between 1996–2004 carried out the PFDI-20 questionnaire and POP-Q examination. We collected data on the presence and type of pelvic floor symptoms and its relation to the degree of pelvic organ prolapse (POP) per compartment (≥ stage 2).

**Results:**

We obtained data from 247 women on average sixteen years after hysterectomy, with no prolapse (*n* = 94), anterior prolapse (*n* = 76), posterior prolapse (*n* = 38), both anterior- and posterior prolapse (*n* = 20), and a prolapse involving the vaginal vault (*n* = 19). Of all 153 women with ≥ stage 2 prolapse, 80 (52%) experienced moderate and/or severe symptoms of the PFDI-20. Most frequently reported symptoms by women with POP were uncontrollable flatus, urinary frequency and urge incontinence. Bulging was associated with a prolapse beyond the hymen. 39% Of women without prolapse experienced bothersome pelvic floor symptoms as well. Most often these were stress incontinence, straining to pass stool and incomplete bowel emptying. Women with a history of hysterectomy for prolapse have more pelvic floor symptoms than women who underwent hysterectomy for other indications, regardless of the current presence of POP (57% versus 40%, *p* = 0.009).

**Conclusion:**

In a group of post-hysterectomy women who did not actively seek help, 47% experienced problematic pelvic floor symptoms, independent of the presence or absence of an anatomic POP. Creating more knowledge and awareness of the impact of hysterectomy on the pelvic floor can help women in the future.

**Trial registration:**

The study was registered in the Dutch Trial Registry; Trial NL5967 (NTR6333, 2017–02-01) and approved by the Medical Research Ethics Committee of the Máxima Medical Center (NL60096.015.16, 2017–02-24).

**Supplementary Information:**

The online version contains supplementary material available at 10.1186/s12905-023-02286-3.

## Background

Pelvic organ prolapse (POP) is a common problem amongst women with a history of hysterectomy, since this procedure is positively correlated to POP [1–3]. In a cohort study of women 16 years after hysterectomy for various indications, 62% of women had POP in at least one compartment, but only 11% had a combination of POP with bulging [4].

Feeling a bulge is the most relevant symptom in detecting a prolapse [5], but it would be incorrect to call POP asymptomatic in the absence of a bulging sensation. POP can cause other pelvic floor symptoms (PFS) as well [6–8]. A significant relationship was demonstrated between posterior prolapse and bowel symptoms, and urinary symptoms and anterior prolapse—as well as POP in general [6, 8].

Pelvic floor symptoms can however be multifactorial, aspecific, increasing by age, and are therefore not always related to POP [6, 9, 10]. Some women with POP do not experience any complaints, while others present with a variety of PFS which severely affect quality of life [8]. The relation between POP and PFS has not been researched in the post-hysterectomy population, where the anatomy is different, and supportive ligaments and vascularisation are affected [10, 11]. However, outcome of this research would give helpful information in order to predict if surgical reconstruction would resolve the symptoms a woman presents herself with.

Regardless of the presence of POP, we do not know how many women experience pelvic floor symptoms (PFS) in the long-term after hysterectomy. And if they do experience PFS, what kind of symptoms, to what extent, and what is the relation to POP.

The purpose of this study is to observe and describe the natural course of PFS of women in the long-term after hysterectomy, and to link this to their pelvic floor anatomy. Our objective is to increase understanding of the relationship between PFS and anatomic prolapse in a post-hysterectomy cohort. This knowledge could help clinicians in counselling women undergoing hysterectomy about pelvic floor symptoms in the future, and when to call a POP ‘symptomatic’ after hysterectomy.

## Methods

### Study design and setting

A cross-sectional cohort study was performed, in accordance with the ‘strengthening the reporting of observational studies in epidemiology (STROBE)’ guidelines [12]. Outcome of this cohort has been partially published in the POP-UP study, assessing the prevalence of prolapse after laparoscopic versus vaginal hysterectomy [4]. Inclusion of participants and data collection was performed in 2017 in a single centre teaching hospital in the Netherlands.

### Participants

Women who underwent a vaginal or laparoscopic hysterectomy between 1996 and 2004 in a single teaching hospital were identified from the surgical registry. Women were excluded if they were over eighty years old, if they had a supracervical hysterectomy, if the indication for hysterectomy was malignancy or if they were deceased in the follow-up period.

Eligible women were invited to complete a single survey and a gynaecologic POP-Q examination. Only women who completed both the survey and the POP-Q examination were included. They were divided into five groups according to the anatomy of their pelvic floor: no prolapse, isolated anterior wall prolapse, isolated posterior wall prolapse, combined anterior and posterior wall prolapse and prolapse involving the vaginal vault.

### Outcomes

The survey elicited information about proven risk factors for POP (e.g. body mass index, age, obstetric history [13–15]), history of POP and pelvic floor symptoms, which were quantified using the Pelvic Floor Distress Inventory (PFDI-20) [16]. This questionnaire had a total of 20 questions using 3 sections relating to each respective compartment: Pelvic Organ Prolapse Distress Inventory (POPDI_6), Urinary Distress Inventory (UDI_6) and Colorectal-Anal Distress Inventory (CRADI_8). For each question, patients scored their symptom severity as “none, mild, moderate or severe”. The score per scale varied from 0–100 points and the maximum score of the PFDI-20 was 300 points (maximum symptom severity for each question). We used the PFDI-data as a continuous variable, but also as a dichotomous variable in order to assess clinical relevance (is the symptom bothersome enough to seek help). The dichotomous variable differentiates between none/mild (scored as 0; not clinically relevant) and moderate/severe symptoms (scored as 1; clinically relevant). The PFDI-20 was validated in Dutch [17].

The pelvic floor examination was performed by a qualified physician using the POP-Q classification. The examiner was blinded for information about the surgical history and other results of the questionnaire. A POP was diagnosed when Ba, C or Bp were ≥ stage 2; according to the POP-Q classification [18].

The primary outcome of this study was to determine the presence and type of PFS, and to correlate this to the presence or absence of POP per compartment.

Response bias was estimated using the questionnaires of women who did not attend the POP-Q examination, by comparing the percentage of women with bulging symptoms.

### Ethical approval

This study was approved by Máxima MC’s Medical Ethical Committee in Veldhoven, the Netherlands (24th February 2017, NL60096.015.16).

### Statistical analysis

Data collection and statistical analyses were performed using SPSS 22.0 0 (SPSS Statistics UK, SPSS, Chicago, IL, USA). Descriptive statistics were used. For outcome comparison, the Pearson chi-square test or Fisher’s exact test was used for ordinal variables and the independent T-samples test was used for continuous variables. A p-value < 0.05 was considered statistically significant.

Due to the skewed distribution of the histogram for the PFDI-20 scores (determined by statistical tests of normality), we have chosen to present the median and interquartile range in the tables rather than the mean scores. This way the impact of outliers is minimalized.

More details on the study design and methods can be found in the first publication (the POP-UP study) [4].

## Results

### Study population

**Fig. 1:**
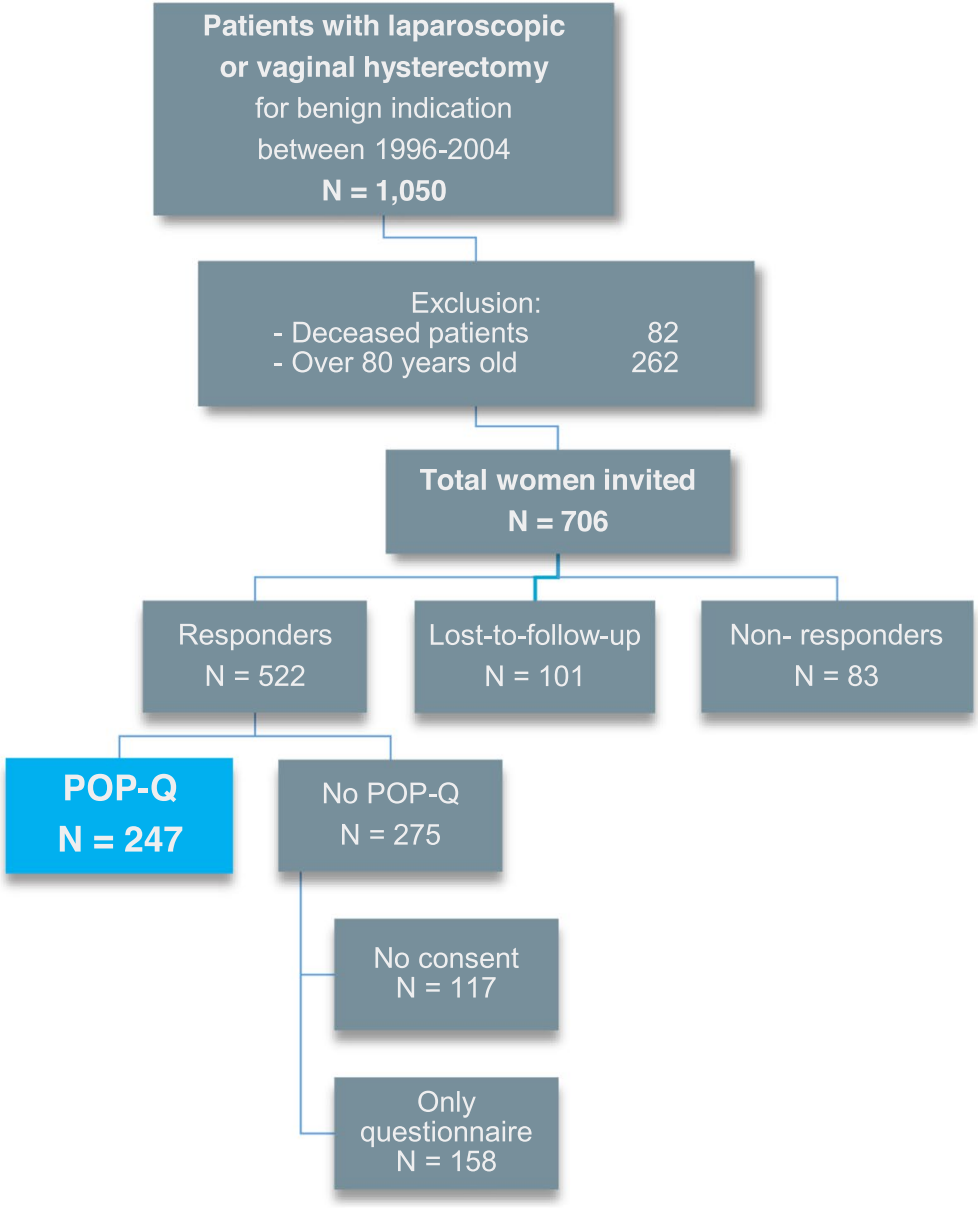
Overview of study design* the included study population is indicated by the blue box

Figure 1 shows the inclusion flowchart. Out of 706 eligible patients, 522 women (74%) responded to the invitation, 101 women (14%) were lost-to-follow-up and 83 women (12%) were non-responders. Of the responders, 247 women (47%) completed the survey and attended the pelvic floor examination; this group was included for this study. 275 responders (53%) refused the POP-Q examination and were therefore excluded. The median follow-up time (interval between hysterectomy and POP-Q examination) was 16 years, ranging from 13 to 21 years. The participant characteristics are presented in Table 1.

**Table 1:** Participant characteristics

Participant characteristics		Total *N* = 247 Number (%) Median (range)
**Age (y)**		64 (45–80)
**Age at hysterectomy (y)**		47 (31–66)
**Body mass index (kg/m2)**		25.7 (19–45)
**Parity (vaginal)**	0	26 (11)
	1	29 (12)
	2	127 (51)
	3 +	65 (26)
**Childbirth weight > 4000 g vaginal**		59 (24)
**Forceps delivery**^**a**^		14 (6)
**Vacuum delivery**^**a**^		15 (6)
**Route of hysterectomy**	Laparoscopic	90 (36)
	Vaginal	157 (64)
**Indication for hysterectomy**	Pelvic organ prolapse	106 (43)
	Abnormal vaginal bleeding	73 (30)
	Myoma	51 (20)
	Abdominal pain	8 (3)
	Endometriosis	5 (2)
	Other^b^	4 (2)
**POP surgery simultaneously with hysterectomy**	None	146 (59)
	Anterior wall	18 (7)
	Posterior wall	7 (3)
	Anterior and posterior wall	76 (31)
	MUS at hysterectomy	3 (1)
**POP treatment after hysterectomy**	None	207 (84)
	Conservative	9 (4)
	Operative	31 (13)
	MUS after hysterectomy	9 (4)

*MUS* Midurethral sling

^a^Two women had one forceps delivery and one vacuum delivery

^b^1x borderline ovarian tumour, 1 × patient request, 2 × cervical dysplasia

Of the women who refused the POP-Q, 200 women were willing to tell us if they had bulging symptoms or not (158 by questionnaire and 42 by phone). This was the case for 7% (*n* = 14) versus 13% of the women included for this study.

Details about age, BMI and obstetric history can be found in Table 1. Ninety women (36%) underwent a laparoscopic hysterectomy and the remaining 157 women had a vaginal hysterectomy (64%). The indication for hysterectomy was pelvic organ prolapse in 43% (*n* = 106) and other benign gynaecological indications for the remaining 57% are listed in Table 1. At time of hysterectomy, 101 women received additional POP surgery simultaneously (41%); in Table 1 the type of procedures is specified.

Forty women received further treatment for POP (16%); nine were conservatively managed (23%) with physical therapy or pessary treatment, and 31 women were treated surgically (77%). Of the 31 patients with further POP surgery, 25 women had a single compartment operation and 6 women received a combined procedure. In total 13 apical procedures, 13 anterior wall repairs and 11 posterior wall repairs were performed. Within the cohort group, 17 women received a midurethral sling for urine incontinence, of which 2 were pre-hysterectomy, 3 during hysterectomy, 9 were post-hysterectomy and for 3 women the timing was unknown.

### Pelvic floor examination and symptoms

All women were examined in 2017. Of the total population (*n* = 247), 153 women (62%) had ≥ stage 2 prolapse in at least one compartment and the other 94 women had prolapse stage 0–1. For the 153 women with a prolapse, 76 women (50%) had an isolated anterior wall prolapse, 38 women (25%) had an isolated posterior wall prolapse, 20 women (13%) had a combined anterior and posterior wall prolapse and 19 (12%) women had a combined prolapse including a ≥ stage 2 vaginal vault prolapse. Numbers of prolapse beyond the hymen are separately displayed in Table 2.

**Table 2:** Overview of results per category of POP ≥ stage 2

	**Isolated anterior wall** ***N* = 76 (%)**	**Isolated posterior wall** ***N* = 38 (%)**	**Combined anterior and posterior wall** ***N* = 20 (%)**	**Prolapse including vaginal vault** ***N* = 19 (%)**	**Any prolapse** ***N* = 153 (%)**	**No prolapse (stage 0–1)** ***N* = 94 (%)**	**Total** ***N* = 247 (%)**
**Above hymen** **(≤ 0)**	65 (85)	36 (95)	13 (65)	2 (10)	116 (76)	94 (100)	210 (85)
**Beyond hymen** **(> 0)**	11 (15)	2 (5)	7 (35)	17 (90)	37 (24)	0 (0)	37 (15)
**Bulging sensation**^**a**^	24 (32)	7 (18)	6 (30)	10 (53)	53 (35)	6 (6)	59 (24)
**PFDI-20 score; median (IQR**^**b**^**)**							
Total	44 (21–78)	37 (23–76)	64 (36–84)	42 (8–73)	44 (23–76)	30 (12–57)	38 (17–69)
POPDI	8 (0–19)	8 (0–17)	17 (0–33)	8 (0–17)	8 (0–21)	4 (0–17)	8 (0–17)
CRADI	9 (5–19)	11 ((3–25)	19 (9–34)	6 (0–25)	9 (3–22)	6 (0–19)	9 (3–22)
UDI	21 (6–44)	17 (4–33)	23 (8–38)	17 (4–25)	21 (8–38)	13 (4–25)	17 (4–33)
**Top 3 symptoms PFDI (only moderate or severe bother)**							
1	Frequent urination (22%)	Uncontrollable flatus (18%)	Uncontrollable flatus (30%)	Bulging sensation (16%)	Uncontrollable flatus (17%)	Stress incontinence (16%)	Uncontrollable flatus (14%)
2	Urge incontinence (20%)	Incomplete bowel emptying (16%)	Pain or discomfort in pelvic area (30%)	Urgency before bowel movement (16%)	Frequent urination (17%)	Straining to pass stool (11%)	Frequent urination (14%)
3	Incomplete bladder emptying (17%)	4 items on third place^c^	Straining to pass stool (25%)	2 items on third place^d^	Urge incontinence (14%)	Incomplete bowel emptying (10%)	Stress incontinence (13%)

^a^Mild, moderate and/or severe bulging sensation according to PFDI-20

^b^IQR = interquartile range; 25^th^ and 75^th^ percentile

^c^13%: Digital evacuation of faeces, incomplete defecation, frequent urination, urge incontinence

^d^11%: Manual micturition, difficulty passing stool

Most patients had a mild prolapse above the hymen; of all ≥ stage 2 prolapses, 24% passed the hymen (Ba, Bp and/or C at + 1 and further). The symptom of feeling a bulge was most common in the group which had vaginal vault involvement; in this group the majority had a prolapse beyond the hymen (90%); of which 82% had a stage 3–4 prolapse (14 out of 17 women).

The scores for all three scales (POPDI, CRADI and UDI) were highest in the group with combined anterior and posterior wall prolapse, with a median score of 64. Six percent of the women without POP still felt a vaginal bulge.

Overall, women with prolapse report higher PFDI-scores in all three sections than women without prolapse, statistically significantly different for the total PFDI-20 score (*p* = 0.016) and POPDI (*p* = 0.022). Micturition symptoms occur most frequently, with an overall median UDI-score of 17 points, versus 8 and 9 points respectively for POPDI and CRADI.

The top three symptoms per prolapse category (Table 2) appears to be associated with the compartment affected. We calculated the correlation of the specific compartment questions of the PFDI-20 with the corresponding prolapse (anterior wall prolapse and UDI-6, posterior wall prolapse and CRADI-8 and vaginal vault prolapse and POPDI-6). An anterior wall prolapse was positively correlated to moderate/severe symptoms for one or more UDI symptoms (*p* = 0.039). This correlation was not found in women with posterior prolapse (*p* = 0.086) or vaginal vault prolapse (*p* = 1.000). In the Additional file 1, an overview of all reported PFDI-20 items is displayed per prolapse compartment. From this table we can see that all PFDI-20 items occur for all types of POP and no evident symptom pattern can be defined.

**Fig. 2:**
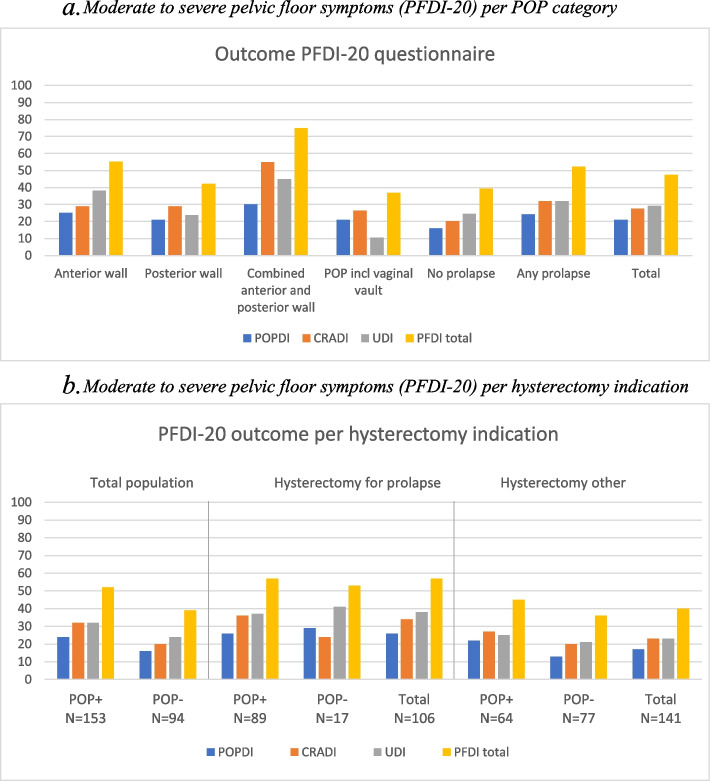
**a** Moderate to severe pelvic floor symptoms (PFDI-20) per POP category. **b** Moderate to severe pelvic floor symptoms (PFDI-20) per hysterectomy indication. **a** and **b** shows the percentage of women with moderate or severe symptoms for at least one item, per POP compartment (≥ stage 2). (n) In (**b**) the participants are categorized according to hysterectomy indication and subcategorized by the presence or absence of any stage ≥ 2 POP at POP-Q examination (POP + and POP-)

We also analysed the PFDI-20 as a dichotomous outcome, splitting the answers into clinically relevant (moderate or severe symptoms) versus not clinically relevant (none or mild symptoms). In Fig. 2a and b, the percentage of women with at least one clinically relevant PFDI-20 item is illustrated for the different groups.

Figure 2a shows that 47% of the population reports at least one clinically relevant pelvic floor complaint. This percentage is 39% for women without POP and 52% for women with any POP, increasing to 75% of women with a combined anterior and posterior wall prolapse (Pearson chi^2^ test *p* = 0.020). Remarkably, the scores of women with POP involving the vaginal vault are comparable to the scores of women without prolapse.

Figure 2b shows the same variable, but women are divided based on their indication for hysterectomy and the presence of anatomic POP. In the group of women who had a hysterectomy for prolapse (*n* = 106), more women have bothersome PFS compared to women with a hysterectomy for other indications (*n* = 141) regardless of the presence of POP (chi^2^ *p* = 0.009).

We did a more detailed analysis of the vaginal vault prolapse group. This group had the highest number of women with POP beyond the hymen, but with minimal symptoms which closely resembles the group without prolapse. Of the 19 women with a POP involving the vaginal vault, 12 women had an apical prolapse beyond the hymen (stage 2: *N* = 2, stage 3–4: *N* = 10). For the remaining 7 women, 5 had an anterior wall prolapse beyond the hymen and 2 did not have a prolapse beyond the hymen. Within the vaginal vault prolapse group, 37% experienced at least one clinically relevant PFS, versus 39% of the group without prolapse (*p* = 0.838). The mean PFDI-20 scores are not significantly different: 44 points in the vaginal vault POP group versus 40 in the no POP group (*p* = 0.619, independent samples t-test). The median scores do differ (vaginal vault 42 points vs no POP 30 points) which indicates that the mean score of the group without prolapse is influenced by outliers.

In order to specify which symptoms are specific for POP, we analysed the difference in prevalence of PFDI-items between women with a POP beyond the hymen (*n* = 37) versus the rest (*n* = 210) using the Chi^2^ test and Fisher’s exact test. The symptoms of feeling a bulge (*p* = 0.024) and having to manually complete micturition (*p* = 0.048) occur significantly more frequently in the POP group, whereas urinary leakage occurs more frequently in the no POP group (*p* = 0.040). Other symptoms were not significantly different between the two groups.

Response bias was estimated using the percentage of women reporting bulging symptoms. In the total population 13% of women experienced bulging. This was only 7% for women who did not participate in the POP-Q examination [4].

## Discussion

### Main outcomes

In this post-hysterectomy cohort that did not seek medical attention, PFS are very common. In total, 47% of women post-hysterectomy reported at least one impacting complaint. Pelvic floor problems are not always related to the presence of POP; women with prolapse only report slightly higher rates of PFS compared to women without POP. Most frequently reported symptoms for women with POP differ according to the affected compartment. The three most common symptoms reported by women without POP are stress urinary incontinence, straining to pass stool and incomplete bowel emptying. A history of hysterectomy for prolapse correlates with more PFS in the long-term compared to women undergoing hysterectomy for other indications, regardless of the presence of anatomic recurrence. A vaginal vault prolapse occurs least commonly and is least symptomatic compared to other prolapse compartments. Only a POP beyond the hymen was related to feeling a bulge. Women with a mild (stage 2) anterior and/or posterior prolapse experienced other kinds of pelvic floor symptoms.

### Interpretation

Several theories about the cause of the increased risk of POP after hysterectomy have been described. The most important one is that by removing the uterus, supporting ligaments are harmed, which lead to more laxity in the pelvic floor [3, 10, 19].

But about PFS after hysterectomy, regardless of POP, not much has been published. There is a hypothesis that due to ligation of the uterine artery, women are more prone to vaginal atrophy. The combination of increased laxity and vaginal atrophy after hysterectomy, and the attempt to (over)compensate by pelvic floor muscle tension, can lead to a variety of PFS [10, 19]. The PFS of women in our study might be (partially) explained as a long-term result of these theories.

In our results section, we decided to show both the absolute PFDI-scores per POP category and the dichotomous variant differentiating between none/mild and moderate/severe symptoms. It is important to point out that you can have a high PFDI-20 score without experiencing moderate or severe symptoms. The PFDI-20 survey was originally designed as a tool to evaluate treatment success, comparing scores before and after treatment [16, 20]. Therefore, no threshold or definition was determined to differentiate clinically relevant PFS. Several validation studies of the PFDI-20 show the use of a mean baseline score (pre-treatment) of 94–122 points when opting for surgery [16, 21] and 60 points when opting for conservative treatment [22]. An arbitrary threshold for clinical relevance could be set at 60 points, however, this is unvalidated. Additionally, a woman with a severe, debilitating symptom linked to only one item would score below this threshold. In order to give clinical relevance to the PFDI score, which is easy to interpret for physicians, we converted the PFDI-20 data into a dichotomous outcome; defining moderate/severe symptoms as clinically relevant.

In our cohort, 47% of women had one or more moderate/severe pelvic floor symptom. Most frequently reported symptoms were uncontrollable flatus, urinary frequency and stress urinary incontinence. In other studies, researching random samples, these symptoms occur frequently as well [8, 17]. Conservative treatment can easily manage some of these symptoms. Are women aware of these treatment options, or have they just simply accepted their symptoms? Perhaps they do not have faith in medical solutions or find it hard to talk about this subject. It would be interesting to know why they did not seek medical help.

We have shown that PFS are very common regardless of whether pelvic organ prolapse is present. For women without POP, 39% had at least one clinically relevant PFS. Sixteen percent (1 out of 6) had a POPDI complaint. A noteworthy finding is shown in Fig. 2b; women who underwent a hysterectomy for POP report more PFS than women undergoing hysterectomy for any other indication, regardless of the current presence of POP (*p* = 0.009). Possibly women with previous POP are more aware of pelvic floor problems, as they may have experienced these symptoms before. Alternatively, they may have a higher risk of urethral mobility and stress urinary incontinence due to pre-existing pelvic floor weakness [23]. These results show us that PFS are multifactorial. Other contributing factors like pelvic floor overactivity, muscle hypertonia or sensory impairment should be recognized to optimize the treatment of pelvic floor dysfunction [24, 25].

We found a significant correlation between women with an anterior wall prolapse and urinary symptoms, consistent with the findings of Slieker et al. [8]. This relationship was not found for bowel and general POP symptoms. This could be explained due to constipation related symptoms frequently being reported by women without POP. This finding was also described by Digesu [6], with the important point that women with constipation are at higher risk of developing POP later in life.

POP is more common after hysterectomy, but we wonder if pelvic floor complaints are too. Unfortunately, PFDI-data for women who did not seek medical attention for pelvic floor dysfunction is scarcely available in literature. Utomo [17] presented the mean PFDI score of a reference group; a representative panel of the Dutch female population (*N* = 283, mean age 47 years, mean parity comparable to our cohort, details about medical history were unknown). The mean PFDI-20 score in this group was 27 points (SD 31), versus 47 points (SD 39) in our post-hysterectomy cohort (mean age 64 years). The lower PFDI-20 score in the reference group suggests a trend towards more PFS after hysterectomy. Unfortunately, due to not having access to medical data and the discrepancy in age, we cannot adequately compare these groups.

PFS can lead to impairment in physical, social and daily activities, and be a real burden for women [10]. Besides adequate help for women with PFS, prevention should be a priority as well. Preventive measures such as lifestyle habits, physical activity, managing constipation and physical therapy are extensively described in the NICE guideline “Pelvic floor dysfunction: prevention and non-surgical management” [26]. We advise to address this to all women opting for hysterectomy.

### Strengths and limitations

This study presents new data regarding pelvic floor symptoms and their relation to pelvic organ prolapse in a random post-hysterectomy cohort. POP-Q data and PFDI-20 data of random samples are lacking in literature, but are necessary to correctly inform patients of the long-term perspective after hysterectomy. Due to our study population not seeking medical help, our data could be interpreted as a representative sample of the general population and could be used for epidemiological purposes.

This study has several limitations. Insight into response bias showed a higher percentage of symptomatic participants (bulging in 13%) versus non-participants (7%). The size of the population is small after dividing participants into subgroups. As mentioned before, we do not have a non-hysterectomy control group, therefore we cannot draw specific conclusions about the influence of hysterectomy on pelvic floor dysfunction in the long-term.

We encountered difficulty in comparing our results to other studies. No previously published data was compatible for comparison due to differences in setting and differences in outcome scales. Most studies did not apply a cut-off for clinical relevance. This can be interpreted as a weakness, but also as a strength given the unique character of our study.

## Conclusion

This study shows a representative picture of pelvic floor symptoms in the long-term after hysterectomy. It is important to note that pelvic floor dysfunction is multifactorial and is not always related to the presence of pelvic organ prolapse. Furthermore, treating the prolapse will not always resolve the symptoms. Even in the non-medical-care-seeking cohort, there is a high frequency of severe pelvic floor symptoms. We consider optimal pelvic floor management to include increasing awareness about prevention of POP, PFS and (conservative) treatment options amongst women at time of hysterectomy.

## Supplementary Information

**Additional file 1:** **Table 3.** Pelvic floor complaints profile per POPcategory.

## Data Availability

The datasets used and/or analysed during the current study are available from the corresponding author on reasonable request.

## Abbreviations

PFS: Pelvic floor symptoms
POP: Pelvic organ prolapse
PFDI: 20 Pelvic Floor Distress Inventory
POP: Q Pelvic organ prolapse quantification
POPDI: 6 Pelvic Organ Prolapse Distress Inventory
UDI: 6 Urinary Distress Inventory
CRADI: 8 Colorectal-Anal Distress Inventory
