# Supplementary material for: Long-term pelvic floor symptoms and urogenital prolapse after hysterectomy
Source: BMC Womens Health. 2023 Mar 21;23:115. doi: 10.1186/s12905-023-02286-3 (PMC10029236; doi:10.1186/s12905-023-02286-3)
Supplement: Supplementary file 1 — Additional file 1: Table 3. Pelvic floor complaints profile per POPcategory. [file 12905_2023_2286_MOESM1_ESM.docx]

Additional file 1: Table 3. Pelvic floor complaints profile per POP category

| PFDI-20 items | Isolated anterior wall | | Isolated posterior wall | | Combined anterior + posterior wall | | Prolapse incl vaginal vault | | Any prolapse | | No prolapse | |
| --- | --- | --- | --- | --- | --- | --- | --- | --- | --- | --- | --- | --- |
|  | N=76 | % | N=38 | % | N=20 | % | N=19 | % | N=153 | % | N=94 | % |
| 1. Experience pressure in the lower abdomen? | 4 | 5% | 1 | 3% | 3 | 15% | 1 | 5% | 9 | 6% | 4 | 4% |
| 2. Experience heaviness or dullness in the pelvic area? | 2 | 3% | 1 | 3% | 2 | 10% | 0 | 0% | 5 | 3% | 3 | 3% |
| 3. Have a bulge or something falling out in your vaginal area? | 4 | 5% | 0 | 0% | 2 | 10% | 3 | 16% | 9 | 6% | 1 | 1% |
| 4. Ever have to push on the vagina or around the rectum to have or complete a bowel movement? | 6 | 8% | 5 | 13% | 3 | 15% | 1 | 5% | 15 | 10% | 6 | 6% |
| 5. Usually experience a feeling of incomplete bladder emptying? | 13 | 17% | 4 | 11% | 0 | 0% | 1 | 5% | 18 | 12% | 6 | 6% |
| 6. Ever have to push on a bulge in the vaginal area to start or complete urination? | 2 | 3% | 0 | 0% | 0 | 0% | 2 | 11% | 4 | 3% | 0 | 0% |
| 7. Feel you need to strain to pass stool? | 5 | 7% | 6 | 16% | 5 | 25% | 2 | 11% | 18 | 12% | 10 | 11% |
| 8. Feel you have not completely emptied your bowels at the end of a bowel movement? | 4 | 5% | 5 | 13% | 1 | 5% | 1 | 5% | 11 | 7% | 9 | 10% |
| 9. Loose stool beyond your control if your stool is well formed? | 2 | 3% | 1 | 3% | 1 | 5% | 0 | 0% | 4 | 3% | 1 | 1% |
| 10. Loose stool beyond your control if your stool is loose? | 6 | 8% | 1 | 3% | 2 | 10% | 1 | 5% | 10 | 7% | 2 | 2% |
| 11. Loose gas from the rectum beyond your control? | 12 | 16% | 7 | 18% | 6 | 30% | 1 | 5% | 26 | 17% | 8 | 9% |
| 12. Have pain when you pass you stool? | 1 | 1% | 1 | 3% | 1 | 5% | 1 | 5% | 4 | 3% | 2 | 2% |
| 13. Experience a strong sense of urgency to have a bowel movement? | 10 | 13% | 2 | 5% | 4 | 20% | 3 | 16% | 19 | 12% | 5 | 5% |
| 14. Does part of your bowel ever pass through the rectum and bulge outside during or after a bowel movement? | 0 | 0% | 2 | 5% | 0 | 0% | 0 | 0% | 2 | 1% | 1 | 1% |
| 15. Experience frequent urination? | 17 | 22% | 5 | 13% | 3 | 15% | 1 | 5% | 26 | 17% | 8 | 9% |
| 16. Experience urine leakage associated with a feeling of urgency? | 15 | 20% | 5 | 13% | 2 | 10% | 0 | 0% | 22 | 14% | 7 | 7% |
| 17. Experience urine leakage related to coughing, sneezing or laughing? | 12 | 16% | 4 | 11% | 2 | 10% | 0 | 0% | 18 | 12% | 15 | 16% |
| 18. Experience small amounts of urine leakage? | 11 | 14% | 3 | 8% | 0 | 0% | 0 | 0% | 14 | 9% | 8 | 9% |
| 19. Experience difficulty emptying your bladder? | 10 | 13% | 4 | 11% | 1 | 5% | 1 | 5% | 16 | 10% | 5 | 5% |
| 20. Experience pain or discomfort in the lower abdomen or genital region? | 8 | 11% | 2 | 5% | 6 | 30% | 0 | 0% | 16 | 10% | 6 | 6% |

**Women with moderate/severe bother*
